# Hybrid GWO-PSO based optimal placement and sizing of multiple PV-DG units for power loss reduction and voltage profile improvement

**DOI:** 10.1038/s41598-023-34057-3

**Published:** 2023-04-27

**Authors:** Assen Beshr Alyu, Ayodeji Olalekan Salau, Baseem Khan, Joy Nnenna Eneh

**Affiliations:** 1 School of Electrical and Computer Engineering, Dilla University, Dilla, Ethiopia; 2 Department of Electrical/Electronics and Computer Engineering, Afe Babalola University, Ado-Ekiti, Nigeria; 3 Department of Electrical and Computer Engineering, Hawassa University, Hawassa, Ethiopia; 4 Department of Electronic Engineering, University of Nigeria, Nsukka, Nigeria; 5 Saveetha School of Engineering, Saveetha Institute of Medical and Technical Sciences, Chennai, Tamil Nadu India

**Keywords:** Engineering, Energy infrastructure, Renewable energy

## Abstract

Distributed generation (DG) is integrated in a passive distribution system to reduce power loss, improve voltage profile, and increase power output. To reap the most benefits of the distribution system, the best location and appropriate DG size must be determined. This paper presents a hybrid Grey wolf Optimizer (GWO) and Particle swarm optimization (PSO) approach for determining the best placement and DG size while considering a multi-objective function that includes active and reactive power loss minimization as well as voltage profile enhancement. Dilla distribution system was used as a case study and the weighted technique was used to convert to a single objective function while taking into account multiple constraints such as bus voltage limit, DG output limit, and branch current limit. DG penetration is limited to up 60% of the total active load on the feeder and a forward–backward sweep load flow algorithm was used to generate the load flow solutions. The findings of the study show that combining three PV-DGs (Case 3) is the best way to improve voltage profile and minimize losses. In addition, the proposed hybrid GWO-PSO algorithm performed better compared to the other four algorithms (Grey Wolf Optimization (GWO), Whale Optimization Algorithm (WOA), Particle swarm optimization (PSO), and sine cosine algorithm (SCA)) in terms of achieving the best multi-objective function (MOF) outcome.

## Introduction

A power distribution network is used to supply power to various customers. Power distribution presents a number of challenges which include high power losses and voltage deviation. Different solutions have been proposed in literature to optimize the performance of a distribution network^1–3^. Presently, Ethiopia’s aggregated power loss is 18.655%, which includes transmission loss, distribution loss, and loss due to power theft^2^. The main challenges that limit the performance of a distribution system are high power loss, low voltage profile, timing and frequency of interruptions, voltage and current harmonic distortions, and unstable voltage in the node of the system^3^. As a result, there is a need to improve the performance of the distribution system in order to make it more reliable and secure. Network reconfiguration, capacitor placement, DG integration, and the incorporation of a FACTS device are some of the techniques for improving the distribution systems performance^4^. The existing radial distribution system (RDS) is passive which means power flows in only one direction from the source to the end node. Network reconfiguration is performed in a distribution network by using tie and sectionalized switches^5^. Distributed Generation (DG) is the installation of small-scale power generation units near load centers that inject active, reactive, or both power into a distribution system. The integration of distributed generation (DG) into a distribution system has numerous advantages, which include reduced power loss, improved voltage profile, and increased system reliability^6^. The term "Distributed Generation (DG)" refers to small-scale electric power generation near the load side (typically 1 kW–50 MW)^7^. To achieve the maximum benefit of DG integration, DGs can be optimally located and sized in a distribution system. Improper placement and sizing of DGs leads to higher power loss and unreliability of the distribution system. There are five common methods for optimizing the placement and sizing of DG and FACTS devices. These are: Analytical, artificial neural network (ANN)-based, meta-heuristic, sensitivity approaches, and combination of sensitivity approaches and meta-heuristic approaches^8–10^. Other methods have been used in literature, but we have used a hybrid of Grey wolf Optimizer (GWO) and Particle swarm optimization (PSO) method. The main advantage of PSO is that there are fewer parameters to configure. In a high-dimensional search field, PSO finds the optimal solution through particle interaction, but it converges to the global optimum very slowly. In addition, it produces low-quality results for complex and large datasets. The Whale Optimization Algorithm (WOA), a swarm-based metaheuristic optimization technique (MOT) inspired by the foraging habits of humpback whales, has so far yielded promising results. However, the WOA, like all MOTs, has drawbacks. Some of these disadvantages include a slow rate of convergence and a limited capacity for exploitation. GWO algorithm is simple in principle, fast in terms of speed, has high search precision, and easy to realize, also has better exploitation ability and is easily combined for use in practical engineering problems. Our proposed algorithm combines the advantages of the two algorithms, which means that PSO has a higher exploration rate and GWO has a higher exploitation rate.

### Literature review

In literature, various researchers have conducted research on optimal placement and sizing of DG in a distribution network for the purpose of achieving technical, economic and environmental benefits.

In Ref.^11^, the authors proposed a flower pollination algorithm for optimal siting and sizing of PV-base DG for loss minimization. In Ref.^12^, the authors proposed a novel hybrid optimization-based algorithm for both single and multi-objective functions with optimal DG allocation in distribution networks. Authors in Ref.^13^ proposed a network reconfiguration algorithm for reliability enhancement, minimizing power losses and thereafter integrated DG. In addition, loss sensitivity factor (LSF) method was adopted for the best combinations of switches as well as placement of DG for minimization of losses. The proposed algorithm was implemented for an IEEE 33-bus RDS. The authors in Ref.^14^ proposed an objective function to find the optimal size and location of solar PV to improve voltage at all the nodes within permissible limits and to reduce power losses in RDS using the PSO algorithm. In Ref.^15^, the authors presented an analytical method for the optimal placement and sizing of DG to minimize power loss and improve voltage profile with fixed DG size and P-type DG and also used IEEE 33 bus for testing the method. The authors in Ref.^16^ proposed a method which precisely identified the optimal location and sizing of DG using three indexes: the Index Vector Method (IVM), the Voltage Deviation Index (VDI), and the Voltage Stability Index (VSI). The Grey Wolf Optimization (GWO), Whale Optimization Algorithm (WOA), and PSO algorithms were used to optimize DG placement and size for power loss reduction. The proposed method was validated using IEEE 33 and 69 bus systems. In order to optimize voltage profile and lower active power losses, the authors in Ref.^17^ proposed an enhanced PSO method for sizing and positioning of DG units in an electrical power system. The MOHTLBOGWO approach was suggested by the authors in Ref.^18^ for determining the best size and placement of DGs in order to minimize power loss and increase the system reliability. A fuzzy-based approach was used to analyze the problem using both single- and multi-objective optimization. In Ref.^19^, the authors used four alternative algorithms, namely, GWO, WOA, PSO, and Teaching Learning Based Optimization (TLBO) to discover the best location and size for DG. Additionally, a comparison of active strategies for reducing power loss was offered. For the best allocation of several DG types in a RDS, the authors in Ref.^20^ presented a hybrid analytical and sine cosine algorithm (SCA), which uses loss sensitivity factor to condense the search space.

### Research gaps

From the reviewed literature, the optimal placement and size of DG is mostly determined by considering a single objective function on an IEEE standard distribution system, while the effect of multiple DGs was not considered. Some of the literature did not use the Backward forward load flow method which is the best load flow technique for a radial distribution system (RDS). Almost all literatures reviewed didn’t use a practical distribution network for analysis of the effect of different algorithms for the optimal placement and sizing of DGs.

### Contribution of the study

There have been numerous studies on the integration of renewable energy based DG into a distributed network for the purpose of achieving technical, economic, and environmental benefits. The main challenge is how to optimally integrate it. The main contribution of the paper is that the PV-DGs are optimally sized and located using the proposed hybrid GWO-PSO, as well as the optimal number of DG to minimize power loss and improve voltage profile was determined to maintain the equality and inequality constraints. The contribution of this paper is listed as follows:

1. A novel hybrid GWO-PSO technique was proposed for the optimal placement and sizing of multiple DGs in a distribution network.
2. The superiority of the proposed hybrid GWO-PSO to other four applied techniques was experimented and compared.
3. Performance comparison of integrating one (1), two (2), three (3), and four (4)-DG to the distribution network to reduce power loss and improve voltage profile is presented.
4. This study employed a practical utility network for testing the system which is located in Dilla Ethiopia. The distribution network has a high-power loss and poor/low voltage profile.

## Modeling of distribution network and PV-DG

**Figure 1:**
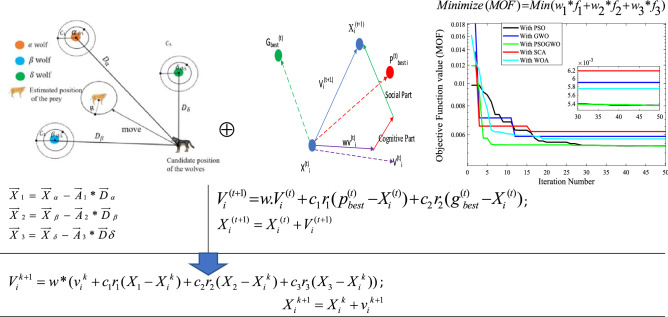
Graphical abstract of the proposed hybrid GWO-PSO based optimal placement and sizing approach.

This section presents the modeling of distribution system including, single line diagram, line data and load data of the feeder and PV-DG modeling. In Fig. 1, the graphical abstract of the proposed approach is presented.

### Distribution network

**Figure 2:**
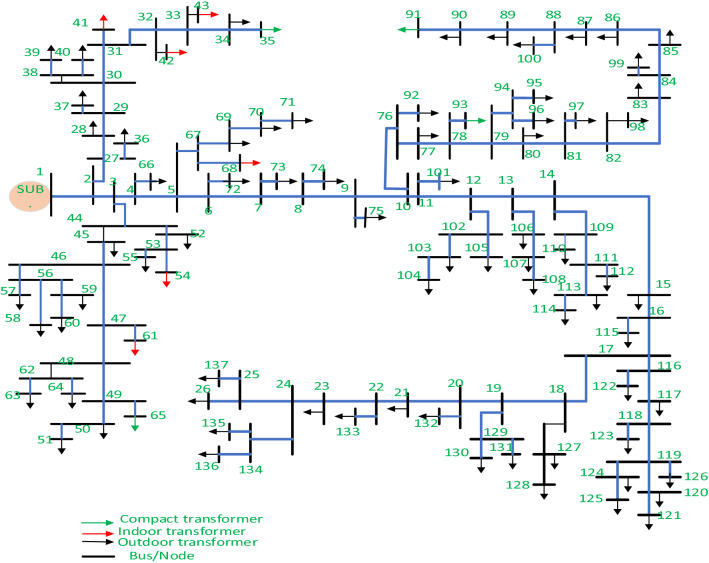
Single line diagram of Dilla one feeder.

The Dilla distribution system was chosen as a case study for the investigation. The distribution substation is located in Gedio zone, South Nation Nationality and People (SNNP) Region, Ethiopia. Three winding power transformers with rated output voltages of 132/633/15 kV, as well as other power system components, make up the distribution substation. Dilla distribution system consists of five outgoing feeders of which three have a rated output voltage of 15 kV and two have a rated output voltage of 33 kV. In this study, only one of the 15 kV feeders which is Dilla one (feeder-1) was considered due to its long-distance coverage which leads to low voltage profile and high-power loss. The Feeder has 137 buses and a total load of 15.793 MW and 9.792 MVAr. Bus one is the slack bus, whereas eighty-three buses are connected to loads with the help of a different sizing distribution transformer and the remaining fifty-three are common coupling nodes as indicated in Fig. 2.

The total length of the overhead distribution line of the Dilla feeder is 30.83 km. The type of conductor used for the distribution lines are stranded conductors of type AAC-50 and Cu-35. These overhead conductors are used to distribute 15 kV voltage from the Dilla substation to the eighty-three distribution transformers. The line impedance is dependent on the length of the sections and the conductor type. The stranded conductors used are AAC 50 mm^2^, where R = 0.5785 Ω/km and X = 0.347 Ω/km, and Cu 35 mm^2^, where R = 0.659 Ω/km and X = 0.374 Ω/km.

The following assumptions are used for the load model.

i. The P & Q of each node is taken as 0.85*kVA and 0.527*kVA of the transformer rating respectively.
ii. The effect of line charging capacitance was neglected due to the short length of the distribution system.

### PV-DG modeling

Load flow analysis of the distribution system considering DG was performed by modeling the DG. The DG is modeled as either constant active and reactive power (P & Q) or constant active power and voltage (PV) model^21^. The P & Q DG model is identified with a constant power load model except that current is absorbed in the load model and injected into a bus for the DG model. Distributed generation is represented in this model as a negative load that changes the direction of the current flow in the radial system (acting as a generator)^22^. Constant active power and constant voltage models have the capability of controlling their reactive power within some limit, to be able to control their voltage within the bus in which they are located. When modeled as a constant active power and constant voltage source, the total reactive power keeps the voltage at a specific value. For this study, PV-DGs are modeled as a constant P & Q model. If P_li_ and Q_li_ are active and reactive power absorbed by the load at bus i before the integration of DG, then after integration, the new active and reactive power absorbed at bus i can be formulated as:

1 $$ P_{nli} = P_{li} - P_{DG} , $$

2 $$ Q_{nli} = Q_{li} - Q_{DG} , $$

where P_nli_ and Q_nli_ are the new real and reactive powers consumed at bus I, respectively.

$P_{DG}$ and $Q_{DG}$ are the real and reactive powers of DG but $Q_{DG}$ is zero because the DG units for this study is solar-based and operating at unity power factor (pf).

## Problem formulation

The backward forward sweep (BFS) approaches utilized for the distribution system power flow analysis, as well as the defined objectives and restrictions, are presented in this section. The goal of this research is to discover the best position and sizing of DG units for the current Dilla-1 system by minimizing the objective functions in real time.

### Distribution system power flow analysis

**Figure 3:**
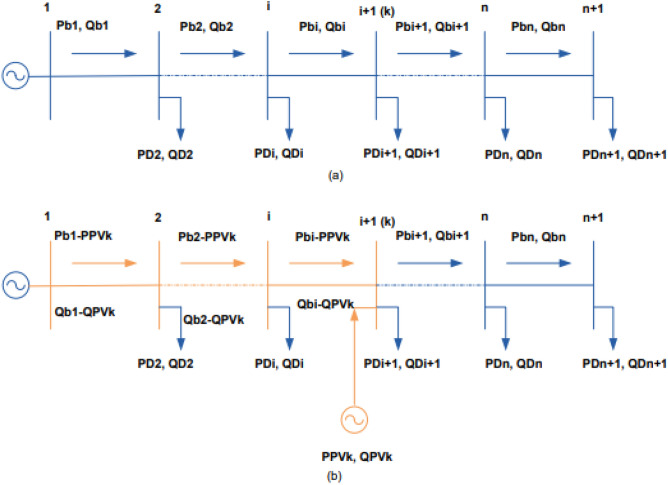
Radial distribution system: (**a**) without PV unit and (**b**) with PV unit.

Consider the following n-branch radial distribution network without a PV unit as shown Fig. 3a. P_bi_ and P_Di_ are the active power flow through branch i and active power demand at the bus i respectively, while Q_bi_ and Q_Di_ are the reactive power flow through branch i and reactive power demand at bus i respectively. In the absence of a PV unit, the total active and reactive power loss (P_L_ and Q_L_) in the distribution system can be calculated using Eqs. (3) and (4) ^23–25^.

3 $$ P_{L} = \sum\limits_{i = 1}^{n} {\frac{{P_{bi}^{2} + Q_{bi}^{2} }}{{\left| {V_{i} } \right|^{2} }}} R_{i} , $$

4 $$ Q_{L} = \sum\limits_{i = 1}^{n} {\frac{{P_{bi}^{2} + Q_{bi}^{2} }}{{\left| {V_{i} } \right|^{2} }}X_{i} } , $$

where R_i_ is the resistance of branch i, X_i_ is the reactance of branch I, and $\left| {V_{i} } \right|$ is the voltage magnitude at bus i.

We assumed that the inverter-based PV technology is capable of delivering active power and delivering or consuming reactive power. The relationship between the active and reactive power (P_PVk_ and Q_PVk_) of PV at integrated bus k is given by Eq. (5) ^26^.

5 $$ Q_{{PV_{K} }} = \alpha_{k} P_{{PV_{K} }} , $$

where $\alpha_{k} = \pm \tan (\cos^{ - 1} (pf_{{pv_{k} }} )).$ Its value is positive for the PV unit supplying reactive power and negative for the PV unit consuming reactive power; and $pf_{{pv_{k} }}$ is the operating power factor of the PV unit at bus k.

The active and reactive power flowing from the source to bus k are lowered due to the PV unit's active and reactive power injected at bus k as shown in Fig. 3b ^25^, while the power flow in the remaining sections are unaffected. Accordingly, the active power loss defined by Eq. (3) and reactive power defined by Eq. (4) can be rewritten as follows:

6 $$ P_{{L_{PV} }} = \sum\limits_{i = 1}^{k} {\frac{{(P_{bi} - P_{{PV_{K} }} )^{2} }}{{\left| {V_{i} } \right|^{2} }}} R_{i} + \sum\limits_{i = k + 1}^{n} {\frac{{P_{bi}^{2} }}{{\left| {V_{i} } \right|^{2} }}R_{i} } + \sum\limits_{i = 1}^{k} {\frac{{(Q_{bi} - Q_{{PV_{K} }} )^{2} }}{{\left| {V_{i} } \right|^{2} }}R_{i} + \sum\limits_{i = k + 1}^{n} {\frac{{Q_{bi}^{2} }}{{\left| {V_{i} } \right|^{2} }}R_{i} } } . $$

Substituting Eqs. (3) and (5) into Eq. (6), we obtained Eqs. (7) and (8):

7 $$ P_{{L_{PV} }} = \sum\limits_{i = 1}^{k} {\frac{{P^{2}_{{PV_{k} }} - 2P_{bi} P_{{PV_{k} }} }}{{\left| {V_{i} } \right|^{2} }}} R_{i} + \sum\limits_{i = 1}^{k} {\frac{{\alpha_{k}^{2} P^{2}_{{PV_{k} }} - 2Q_{bi} \alpha_{k} P_{{PV_{k} }} }}{{\left| {V_{i} } \right|^{2} }}} R_{i} + P_{L} , $$

8 $$ Q_{{L_{PV} }} = \sum\limits_{i = 1}^{k} {\frac{{P^{2}_{{PV_{k} }} - 2P_{bi} P_{{PV_{k} }} }}{{\left| {V_{i} } \right|^{2} }}} X_{i} + \sum\limits_{i}^{k} {\frac{{\alpha_{k}^{2} P^{2}_{{PV_{k} }} - 2Q_{bi} \alpha_{k} P_{{PV_{k} }} }}{{\left| {V_{i} } \right|^{2} }}X_{i} } + Q_{L} . $$

For this study, the PV system has unity power factor which means it generates only active power or $\alpha_{k} = 0$.

### Objective function and system constraints

This subsection presents the formulation of the objective function which consists of loss minimization and voltage profile improvement and also system constraints including the equality and inequality constraints.

#### Objective function

The objective functions for this study are power loss minimization and voltage profile improvement.

A. Loss minimization

The feeders total active power losses can be calculated using Eq. (9).

9 $$ f_{1} = \sum\limits_{i = 1}^{nb} {R_{i} \times I_{i}^{2} } . $$

Similarly, the feeders total reactive power losses can be computed using Eq. (10).

10 $$ f_{2} = \sum\limits_{i = 1}^{nb} {X_{i} \times I_{i}^{2} } , $$

where *f*_*1*_ and *f*_*2*_ are the first and second objective functions associated with the system power loss minimization.

I_i_ is the current of line i, Ri is the resistance of the ith line, nb is the number of system branches.

The percentage of total power loss reduction can be calculated using Eqs. (11) and (12).

11 $$ \% \,Active\,loss\,reduction = \frac{{P_{{_{L} }} - P_{{L_{PV} }} }}{{P_{{L_{PV} }} }} \times 100, $$

12 $$ \% \,Reactive\,loss\,reduction = \frac{{Q_{{_{L} }} - Q_{{L_{PV} }} }}{{Q_{{L_{PV} }} }} \times 100. $$

B. Voltage profile improvement

The second objective function is improving the voltage profile of the distribution network which is the commutative voltage deviation index described as follows:

13 $$ f_{3} = \sum\limits_{i = 1}^{N} {(1 - V_{i} )}^{2} . $$

V_i_ is the voltage of the ith bus, N is the number of the system buses.

The formulation of the general multi-objective function (MOF) and its conversion into a single objective using the weight sum method is given by:

14 $$ Minimize\,\,\,(MOF) = Min(w_{1} \times f_{1} + w_{2} \times f_{2} + w_{3} \times f_{3} ), $$

$$ {\text{where}}\,\,\sum\limits_{i = 1}^{3} {w_{i} } = 1. $$

The advantage of using the weighted sum method includes its ease of use, specifically when working with convex problems. Its disadvantage includes not being able to find all solutions in a non-convex solution space and not having a straightforward way of assigning the weights of the objectives. The determination of the proper weighting factors is also dependent on the experience and concerns of the system planner. For this study, our major concern is active power loss due to its impact on utility profit as it accounts for 50% or more of utility profit loss and unsatisfaction of the consumers. Different weight probabilities are tested, and one weight factor combination that provides a minimum objective function as presented in the result section.

#### System constraint

The system constraints are categorized as follows:

A. Equality constraints

The active and reactive power flow in the RDS is included in the equality constraints and are calculated in Eqs. (15) and (16).

15 $$ P_{s} + \sum\limits_{i = 1}^{np} {P_{pv} } = \sum\limits_{h = 1}^{n} {P_{D} (h)} + \sum\limits_{j = 1}^{nl} {P_{loss(j)} } , $$

16 $$ Q_{s} = \sum\limits_{h = 1}^{n} {Q_{D} (h)} + \sum\limits_{j = 1}^{nl} {Q_{loss(j)} } , $$

where,$P_{s} \,and\,Q_{s}$ are the supplied active power and the supplied reactive power at the substation, respectively. $P_{D} \,and\,Q_{D}$ are the active load and reactive load, respectively, while nl is the number of lines in the RDS.

- Inequality constraint
- Bust voltage limits
  17 $$ V_{\min } \le \,V_{i} \, \le \,V_{\max } , $$
  where V_min_ and V_max_ are the lower and the upper voltage limits. Where *V*_min_ = 0.95 pu and *V*_max_ = 1.05 pu.

(b) Total injected active power limit

The active power of the PV should be equal to or less than the active power load. In this case, the maximum limit of the total capacity of DG units is 60% of the total kW loading of the network, while its minimum limit is zero. This can be calculated using Eq. (18).

18 $$ \sum\limits_{i = 1}^{np} {P_{pv} (i)} \le \sum\limits_{i = 1}^{n} {P_{D} } (i), $$

where, P_D_ is the active load and P_pv_ is injected active power of the PV units.

(c) Line thermal units

The line thermal unit can be calculated using Eq. (19).

19 $$ I_{k} \le I_{\max ,k} \,\quad k = 1,2,3 \ldots Nb, $$

where Nb is the number of branches in the network.

### Optimization algorithm

This section describes the fundamental principle and mathematical modeling of the grey wolf optimizer (GWO) and particle swarm optimization (PSO) method and also the proposed hybrid GWO-PSO including the flowchart of proposed method.

#### Grey wolf optimizer

The Grey wolf optimizer (GWO) algorithm was developed by Mirjalili and Lewis^27^. Grey wolves are social animals with strict social hierarchy. There are four types of grey wolves within the leadership hierarchy of the GWO algorithm. These are alpha, beta, delta, and omega wolves. In the GWO algorithm, alpha wolves represent the solution with the best result. Beta and delta wolves represent the second and third best solutions in the population. Omega wolves are the best solution candidates. The mathematical modelling of GWO is based on the social hierarchy and hunting behavior of grey wolves. Grey wolves’ hunting tactics includes the following three main parts: (1) Tracking, chasing, and approaching the prey. (2) Pursuing, encircling, and harassing the prey till it stops moving. (3) Attacking the prey. Encircling the prey is modeled mathematically using the following equations:

20 $$ r = \mathop {\lim }\limits_{x \to \infty } x, $$

21 $$ \overrightarrow {D} = \left| {\overrightarrow {C} *\overrightarrow {X}_{p} (t) - \overrightarrow {X} (t)} \right|, $$

22 $$ \overrightarrow {X} (t + 1) = \overrightarrow {X}_{p} (t) - \overrightarrow {A} *\left| {\overrightarrow {C} *\overrightarrow {X}_{p} (t) - \overrightarrow {X} (t)} \right|, $$

where, ‘t’ is the number of current iterations, ‘Xp’ is the position of the prey, ‘X’ is the location of the grey wolves, and ‘A’ and ‘C’ are the coefficients for the vectors. The coefficients ‘A’ and ‘C’ are calculated using Eqs. (23) and (24).

23 $$ \overrightarrow {A} = \overrightarrow {a} \times (2\overrightarrow {r}_{1} - 1), $$

24 $$ \overrightarrow {C} = 2 \times \overrightarrow {r}_{2} , $$

where, the number of ‘a’ is linearly decreasing from 2 to 0 as the number of iterations decreases. r_1_ and r_2_ represent uniformly selected random numbers between [0, 1]. In the hunting process of grey wolves, alpha is considered the optimal applicant for the solution, while beta and delta are expected to be knowledgeable about the prey’s possible position. Therefore, the three best solutions that are achieved are kept until a certain iteration which forces others (e.g., omega) to modify their positions in the decision space consistent with the best place. The position is updated as follows:

25 $$ \overrightarrow {X} (t + 1) = \frac{{\overrightarrow {X}_{1} + \overrightarrow {X}_{2} + \overrightarrow {X}_{3} }}{3}, $$

where X_1_, X_2_, X_3_ are calculated as follows:

26 $$ \begin{gathered} \overrightarrow {X}_{1} = \overrightarrow {X}_{\alpha } - \overrightarrow {A}_{1} \times \overrightarrow {D}_{\alpha } \hfill \\ \overrightarrow {X}_{2} \, = \overrightarrow {X}_{\beta } - \overrightarrow {A}_{2} \times \overrightarrow {D}_{\beta } \hfill \\ \overrightarrow {X}_{3} \, = \overrightarrow {X}_{\delta } \, - \overrightarrow {A}_{3} \, \times \overrightarrow {D} \delta . \hfill \\ \end{gathered} $$

The values $X_{\alpha } ,X_{\beta } ,\,\,{\text{and}}\,X_{\delta }$ represent the best three wolves in each iteration, respectively. Where, *A*_1_, *A*_2_, and *A*_3_ are calculated as in Eq. (26). $D_{\alpha } ,D_{\beta } ,D_{\delta }$ are calculated as in Eq. (27).

27 $$ \begin{gathered} \overrightarrow {D}_{\alpha } = \left| {\overrightarrow {C}_{1} \times \overrightarrow {X}_{\alpha } - \overrightarrow {X} } \right| \hfill \\ \overrightarrow {D}_{\beta } = \left| {\overrightarrow {C}_{2} \times \overrightarrow {X}_{\beta } - \overrightarrow {X} } \right| \hfill \\ \overrightarrow {D}_{\delta } = \left| {\overrightarrow {C}_{3} \times \overrightarrow {X}_{\delta } - \overrightarrow {X} } \right|, \hfill \\ \end{gathered} $$

where C_1_, C_2_, C_3_ are calculated based on Eqs. (24) and (27). Grey wolves finish their hunting by attacking the prey. To achieve this, they must get close enough to the prey. When Eq. (23) is examined, ‘A’ takes values that vary from [− 2a, 2a], while ‘A’ takes decreasing values from 2 to 0. When |A| value is greater than or equal to 1, the existing hunts are abandoned to find better solutions. Assuming that the prey gets close enough for values less than 1, the grey wolves are forced to attack the prey. This approach prevents the wolves from getting stuck on the local minimum. When the GWO algorithm reaches the desired number of iterations, the search is completed.

#### Particle swarm optimization

Particle Swarm Optimization (PSO) is a population-based algorithm that was developed by R. Eberhart and J. Kennedy in 1995^28^. It was inspired by the movement of organisms such as bird flocking and fish schooling. The randomly generated population is called a swarm and it consist of individuals named particles. Every particle in the swarm indicates a probable explanation of the optimization problem. Each particle moves with a random velocity through a D-dimensional search space^29,30^. The particle (X_i_) is the position representation of each individual with N-dimensional search space which is described using Eq. (28).

28 $$ X_{i} = (X_{i1} \,X_{i2} \,X_{i3} \ldots X_{iN} ). $$

Then each particle moves to become a new particle position ($X_{i}^{(t + 1)}$) by updating the velocity through a new speed variable ($V_{i}^{(t + 1)}$) with the following equation:

29 $$ V_{i}^{(t + 1)} = w.V_{i}^{(t)} + c_{1} r_{1} (p^{(t)}_{best} - X_{i}^{(t)} ) + c_{2} r_{2} (g^{(t)}_{best} - X_{i}^{(t)} ), $$

30 $$ X_{i}^{(t + 1)} = X_{i}^{(t)} + V_{i}^{(t + 1)} , $$

where C_1_and C_2_ are individual and group acceleration coefficients respectively, r_1_ and r_2_ are random values between [0–1].$w(t)$ is the weight value of the inertia at iteration t, $w(t)$ is calculated using Eq. (31) ^31^.

31 $$ w(t) = \left( {w_{\max } - \frac{{w_{\max } - w_{\min } }}{\max .iter}} \right) \times t. $$

#### Hybrid GWO and PSO

**Figure 4:**
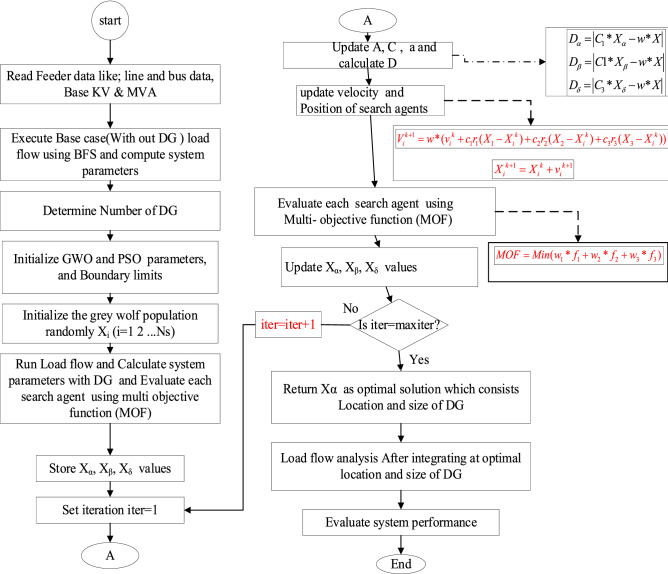
Flowchart of the proposed hybrid GWO-PSO for optimal sizing and location of DG.

In Ref.^32^, the hybrid GWO-PSO algorithm was presented for improving the performance of convergence. Authors have used the GWO-PSO to combine the capacity of both methods and to explore PSO with the ability to exploit GWO in order to reach their optimized strengths^33^. Instead of utilizing the traditional mathematical equations, the first three agents' positions in the search space are updated in the proposed hybrid GWO-PSO, and the grey wolf's exploitation and exploration were governed by inertia constant. The overall flowchart for the optimal placement and sizing of the PV-DGs is shown in Fig. 4.

## Result and discussion 

The analysis and simulation was performed using MATLAB 2021a with an Intel(R) Core (TM) i5-4300U CPU @ 1.90 GHz, 8 GB RAM on a personal computer. By testing different probability of weighting factors as shown in Table 1, we selected w_1_ = 0.5, w_2_ = 0.4, and w_3_ = 0.1 which give a minimum objective value.

**Table 1:** Effect of weights on the fitness value.

w_1_	w_2_	w_3_	Best objective function value
0.5	0.1	0.4	0.0181
0.5	0.2	0.3	0.0145
0.5	0.3	0.2	0.0107
0.5	0.4	0.1	0.007
0.6	0.1	0.3	0.0146
0.6	0.2	0.2	0.011
0.6	0.3	0.1	0.0073
0.7	0.1	0.2	0.0111
0.7	0.2	0.1	0.00734
0.8	0.1	0.1	0.0075

The input parameters of the different optimization algorithms and boundary condition of the decision variables are presented in Table 2.

**Table 2:** Parameters of the optimization algorithm and boundary of decision variables of the objective function.

Parameters of PSO algorithms	Number of particles (No. P) for PSO		30
	Number of search agents for GWO, SCA, and WOA		30
	Maximum iteration		50
	Inertia weight	$w_{\min }$	0.4
		$w_{\max }$	0.9
	Personal best value (c1)		2
	Neighborhood best value (c2)		2
Decision variables of objective function		Bus number (location)	Sizing of DG (MW)
Boundary	Lower bound (Lb)	2	0
	Upper bound (Ub)	137	9.476

In the existing or base case, the total active and reactive load demands are 15.793 MW and 9.792 MVAr respectively. The real power loss of the system is 888.9041 KW, whereas its reactive power loss is 531.4082 kVar at the base case. The minimum voltage is 0.906 p.u at bus number 125 which is below the limit of 0.95pu and also seventy-nine buses are below the required voltage level (57.66%). Multiple PV-DG units are integrated into the system to analyze the performance of the feeder.

The system inputs data like base MVA = 100 and base kV = 15 which are used to change the base value into a per-unit value. Four cases are used for the integration of PV-DG units which are from one up to 4-DG in number by applying five optimization algorithms, namely, PSO, GWO, SCA, WOA, and hybrid GWO-PSO. The four cases are: Case 1: One DG (1-DG) integration; Case 2: Two DG (2-DG) integration; Case 3: Three DG (3-DG) integration; Case 4: Four DG (4-DG) integration.

**Figure 5:**
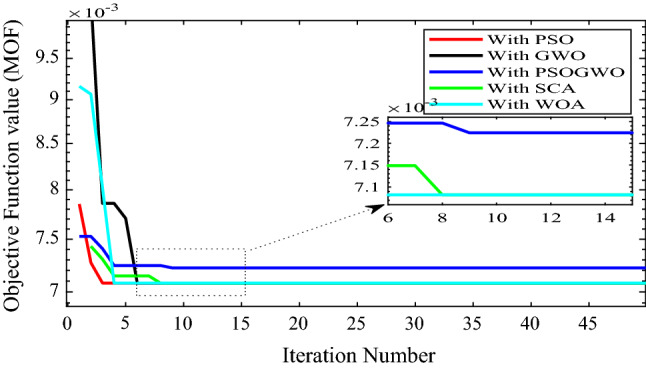
Convergence curve for 1-DG integration with different algorithms.

For case 1, the overall performance measurement is shown in Fig. 5 and Table 3.

**Table 3:** Performance measurement after 1-DG integration with different algorithms.

Methods	Location (size in KW)	P_Loss_ (kW)	Q_Loss_ (kVar)	Vmin (p.u.)	Max. voltage deviation (%)	% P loss reduction	% Q loss reduction	MOF
PSO	17 (9475)	455.9757	271.1165	0.95893	4.1072	48.5914	48.874	0.00723
GWO	17 (9475)	455.9757	271.1165	0.95893	4.1072	48.5914	48.874	0.00723
GWO-PSO	116 (9475)	473.8856	281.2894	0.95884	4.1163	46.5722	46.9556	0.00722
SCA	17 (9475)	455.9757	271.1165	0.95893	4.1072	48.5914	48.874	0.00723
WOA	17 (9475)	455.9757	271.1165	0.95893	4.1072	48.5914	48.874	0.00723

**Figure 6:**
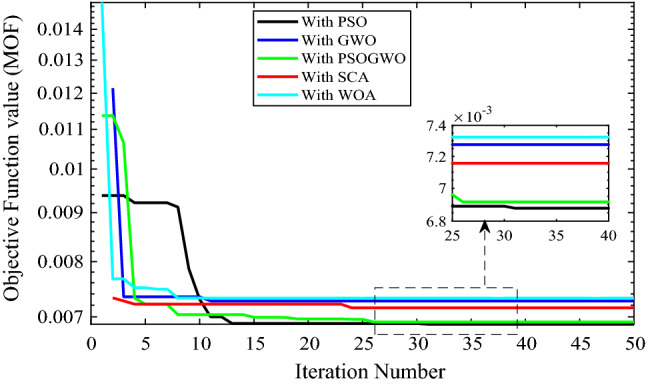
Convergence curve for 2-DG integration with different algorithms.

Figure 6 shows the result obtained when integrating 2-DG with different algorithms. It was observed that each algorithm has a different number of iterations to reach its optimal solution, whereas GWO and WOA converge rapidly with less than 15 iterations. Also, PSO and GWO-PSO converge after 30 iterations and PSO achieves a minimum multi-objective function value. The overall performance is illustrated in Table 4.

**Table 4:** Performance measurement after 2-DG integration with different algorithms.

Methods	Location (size in kW)	P_Loss_ (kW)	Q_Loss_ (kVar)	Vmin (p.u.)	Max. voltage deviation (%)	% P loss reduction	% Q loss reduction	MOF
PSO	14 (4738)	401.735	240.3636	0.95921	4.079	54.7067	54.6732	0.006878
	19 (4738)							
GWO	82 (4738)	442.295	264.6681	0.9705	2.95	50.1339	50.09	0.007275
	137 (4738)							
GWO-PSO	132 (4738)	411.512	246.2272	0.95916	4.0841	53.6044	53.5675	0.00692
	14 (4738)							
SCA	106 (4738)	418.084	250.1675	0.95913	4.0875	52.8635	52.8244	0.007156
	19 (4738)							
WOA	129 (4533)	463.597	275.4597	0.9581	4.19	47.732	48.0549	0.00732
	122 (4738)							

**Figure 7:**
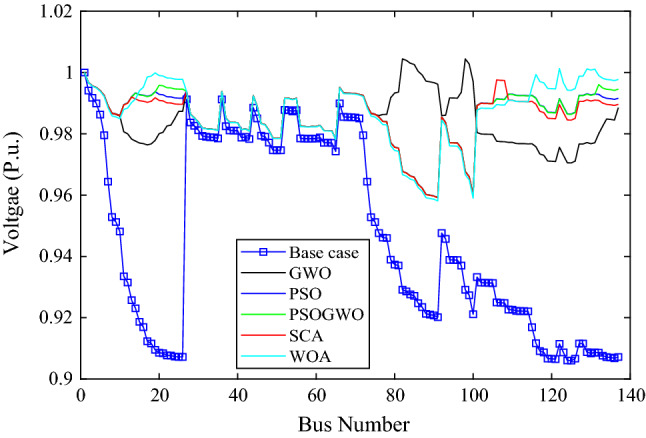
Voltage profile of base case and 2-DG integration with different algorithms.

As shown in Fig. 7, all algorithms converged with few iterations (less than ten) and also the PSO, WOA, SCA and GWO based optimization gives minimum and the same objective function values. While the hybrid GWO-PSO gives higher values. All algorithms except the hybrid GWO-PSO give bus 17 and 9475 kW as the optimal location and size of the DGs. The percentage real and reactive power loss reduction are 48.5814 and 48.874 respectively. Also, the minimum voltage improved from 0.906 p.u to 0.95893 p.u, while the hybrid GWO-PSO achieves bus 16 and 9475 kW as optimal location and size which gives a 46.5722% real and 46.9556% reactive power loss reduction as well as a minimum voltage of 0.95884 p.u.

The voltage profile for 2-DG integration with different algorithms is shown in Fig. 6. The results show that bus 1–7 and bus 27–73 have the same voltage profile for different algorithms. However, other buses have different voltage profiles. The maximum voltage is achieved at bus 82 with a value of 1.00444 p.u using the GWO algorithm, while the minimum voltage is achieved at bus 91 with a value of 0.9581 p.u using WOA. Algorithm like PSO and GWO-PSO have nearly the same voltage profile except bus 20–26 and bus 132 to last bus.

**Figure 8:**
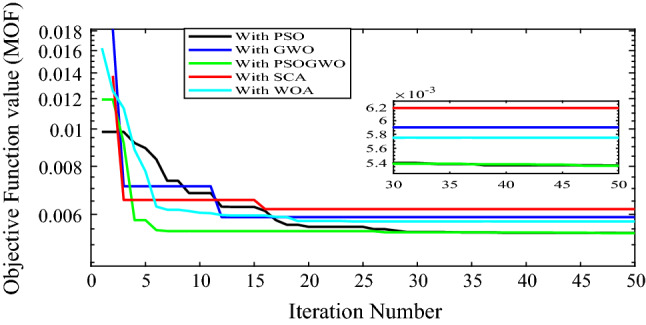
Convergence curve for 3-DG integration with different algorithms.

The convergence characteristics of the different optimization algorithms for the optimal integration of multiple (three) PV-based DG units in the Dilla one distribution system is shown in Fig. 8. Figure 8 shows the results obtained when the different optimization algorithms are applied to the test system. It was observed that each algorithm has a different number of iterations to reach the optimal solution, whereas the GWO, SCA, and hybrid GWO-PSO converge rapidly with less than 18 iterations. In fact, the hybrid GWO-PSO algorithm converges faster compared to the other algorithms, taking less than seven iterations, and having the least value of MOF. Also, it was observed that the PSO and hybrid GWO-PSO algorithms converge with the same value of objective function (0.005388) using the Dilla one feeder test system considered in this paper. The optimization results of three PV-DGs using different optimization algorithms for the Dilla one feeder are tabulated in Table 5.

**Table 5:** Performance measurement after 3-DG integration with different algorithms.

Methods	Location (size in KW)	P_Loss_ (kW)	Q_Loss_ (kVar)	Vmin (p.u.)	Max. voltage deviation (%)	% P loss reduction	% Q loss reduction	MOF
PSO	22 (3156)	365.421	218.0724	0.97827	2.1734	58.8009	58.8768	0.00539
	86 (3156)							
	123 (3156)							
GWO	85 (2880)	390.914	233.0712	0.97814	2.1856	55.9267	56.0484	0.0059
	137 (3156)							
	125 (3156)							
GWO-PSO	86 (3156)	371.4827	221.5613	0.97826	2.1737	58.1175	58.2189	0.00536
	119 (3156)							
	133 (3156)							
SCA	80 (3156)	344.9114	205.9623	0.97177	2.8233	61.1133	61.1605	0.00619
	127 (3156)							
	19 (3156)							
WOA	137 (3156)	385.117	229.741	0.97814	2.1856	56.5803	56.6764	0.00575

**Figure 9:**
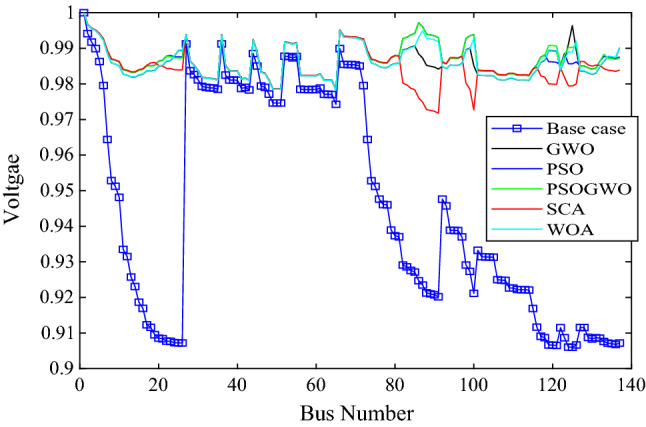
Voltage profile of base case and 2-DG integration with different algorithms.

Figure 9 shows the voltage profiles before and after the integration of 3-DG units into the Dilla one feeder test system. The voltage profiles of bus numbers 1–6 and 27–66 are quite similar for all algorithms. On the other hand, for the rest of buses, there are noticeable variations in the voltage profiles.

**Figure 10:**
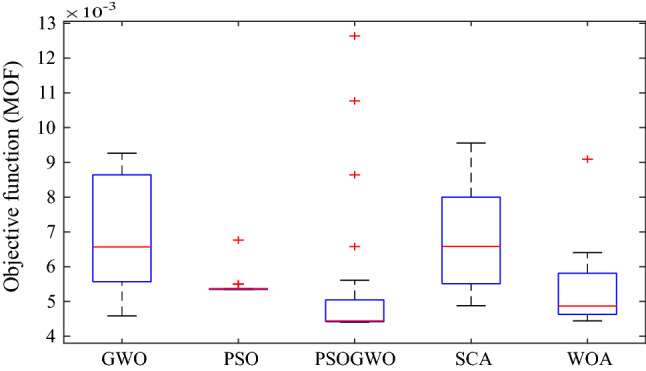
Boxplot of MOF using different algorithms.

Figure 10 shows the boxplot of MOF while using different algorithms for the Dilla one feeder by considering the same parameters such as the number of iterations and population size, and the outcomes of different algorithms after 20 runs. The results show that the hybrid GWO-PSO gives the best result of MOF which gives the minimum median value.

**Figure 11:**
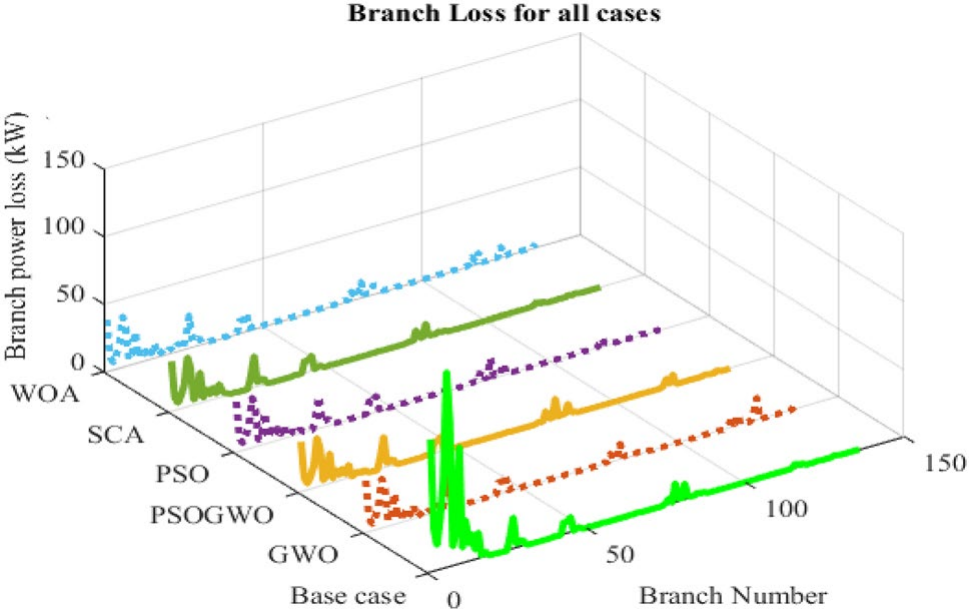
Branch power loss considering different algorithms.

**Figure 12:**
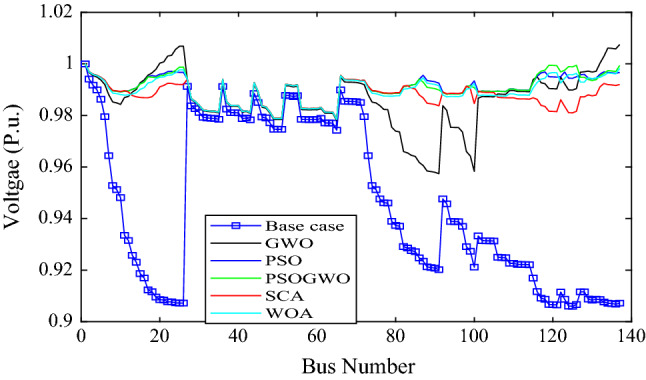
Voltage profile of base case and 4-DG integration with different algorithms.

The voltage profile is different for different algorithms except for bus 27–66 which is nearly the same as shown in Fig. 11. The minimum voltage is 0.9574p.u at bus 91 and the maximum voltage is 1.00749 p.u at bus 137 for GWO in both cases. As shown in Fig. 12, the WOA converges rapidly while it has the highest MOF value, except for the GWO algorithm, while the PSO has best (minimum) MOF value. Table 6 presents the performance measurement after 4-DGs were integrated with different algorithms.

**Table 6:** Performance measurement after the integration of 4-DGs with different algorithms.

Methods	Location (size in kW)	P_Loss_ (kW)	Q_Loss_ (kVar)	Vmin (p.u.)	Max. voltage deviation (%)	% P loss reduction	% Q loss reduction	MOF
PSO	87 (2569)	377.2116	224.8239	0.97857	2.1425	57.4716	57.6036	0.00442
	23 (2569)							
	128 (2569)							
	118 (2562)							
GWO	128 (2336)	478.4918	285.2436	0.95741	4.2595	46.0528	46.2099	0.00770
	137 (2569)							
	115 (1638)							
	134 (2569)							
GWO-PSO	119 (2569)	387.5865	230.7713	0.97857	2.1428	56.3019	56.4821	0.00451
	137 (2569)							
	86 (2569)							
	122 (2569)							
SCA	21 (2569)	353.0925	211.1298	0.97859	2.141	60.1909	60.186	0.00496
	11 (2569)							
	83 (2569)							
	22 (2569)							
WOA	137 (2293)	379.856	226.437	0.97847	2.1532	57.1735	57.2994	0.00462
	120 (2569)							
	87 (2569)							
	129 (2569)							

**Figure 13:**
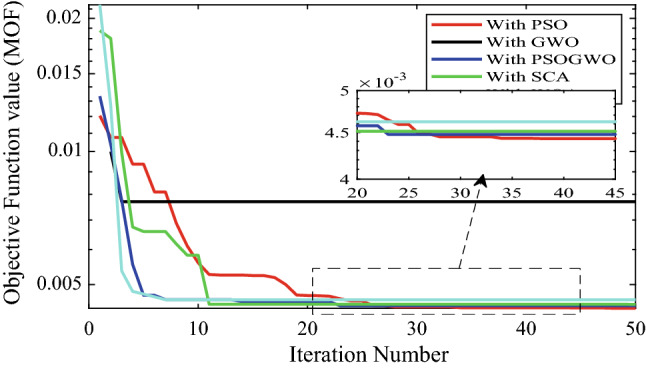
Convergence curve for 4-DG integration with different algorithms.

**Figure 14:**
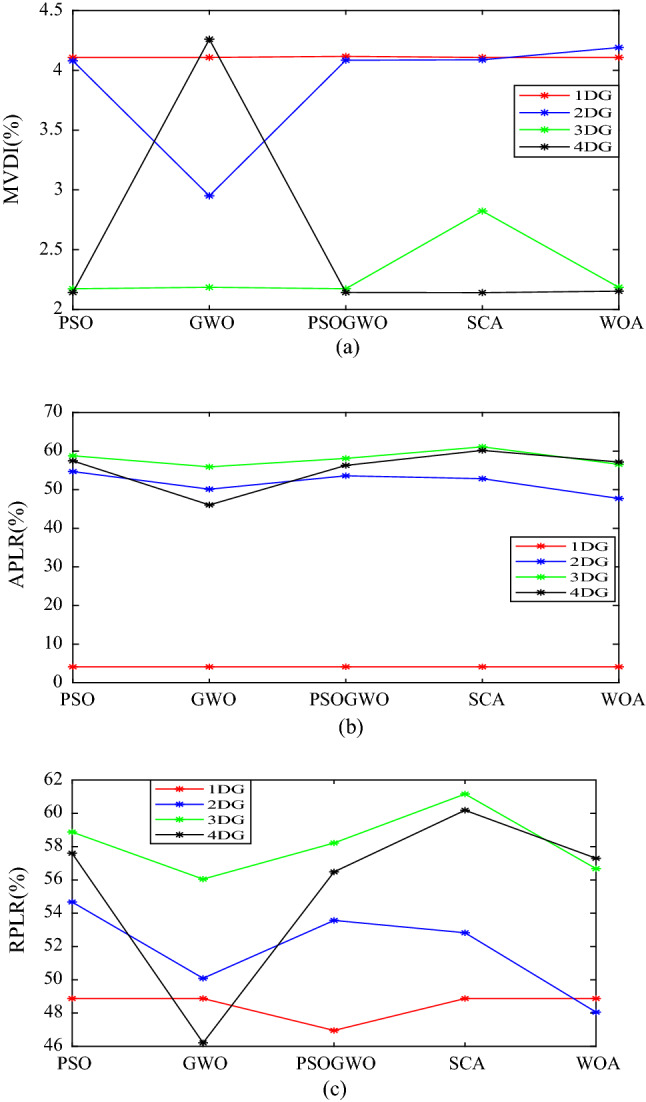
Comparison of different DGs and algorithms (**a**) for MVDI (%), (**b**) APLR (%), (**c**) RPLR (%). *MVDI* Maximum voltage deviation index, *APLR* active power loss reduction, *RPLR* reactive power loss reduction.

The convergence curve for integration of 4-DG with different algorithms is shown in Fig. 13. From Fig. 14, it is observed that 3-DG integration has high percentage of active and reactive power loss reduction and minimum voltage deviation index.

Figure 14 shows that GWO gives the best MVDI (%). This indicates that GWO is suitable for voltage improvement and also PSO gives the best APLR (%) and RPLR (%). This indicates that PSO is suitable for power loss reduction. However, PSO-GWO gives the best result for both voltage improvement and loss reduction.

The proposed hybrid algorithm is not computationally complex as it achieved a runtime (processing time) of 2.78 s, while 2.89 s, 2.9 s, 3.12 s, and 3.2 s was achieved using PSO, GWO, SCA, and WOA, respectively.

## Conclusion and recommendation for future work 

### Conclusion

This paper presented the integration of Multiple PV-DGs to minimize the multi-objective function for active and reactive power loss reduction and voltage profile improvement in an Ethiopian distribution system in Dilla city (Dilla distribution system). The results of the study show that the percentage real and reactive power loss reduction is high at three DG (case-3) almost for all algorithms and less maximum voltage deviation and minimum. In addition, the superiority of the proposed hybrid GWO-PSO algorithm was shown compared to the other four algorithms (Grey Wolf Optimization (GWO), Whale Optimization Algorithm (WOA), Particle swarm optimization (PSO), and sine cosine algorithm (SCA)) in terms of achieving the best result of multi-objective value (MOF). By integrating three DG using the proposed method, 58.1175% active and 58.2189% reactive power loss was reduced, moreover the voltage profile is within permissible limits where the maximum voltage deviation is 2.1739%.

### Recommendation for future work

In the future, authors hope to use other meta-heuristic approaches for the Optimal Placement and Sizing of Multiple PV-DG Units installing battery energy storage systems (BESSs).

## Data Availability

The datasets generated during and/or analyzed during the current study are not publicly available but are available from the corresponding author on reasonable request.
